# Cytokines as Key Drivers of Pathological Root Resorption: Integrating Molecular Mechanisms, Genetic Determinants, and Biomarker-Based Insights

**DOI:** 10.3390/biomedicines14061256

**Published:** 2026-05-30

**Authors:** Romina-Christiana Pavlovici, Cristina-Crenguţa Albu, Claudia Florina Bogdan-Andreescu, Viorica Tudor, Lucia Bubulac, Iuliana-Raluca Gheorghe, Arsenie Dan Spînu, Emin Cadar, Dan Alexandru Slăvescu, Mariana Păcurar

**Affiliations:** 1 Doctoral School, “Carol Davila” University of Medicine and Pharmacy, 020021 Bucharest, Romania; romina-christiana.pavlovici@drd.umfcd.ro; 2 Department of Genetics, Faculty of Dentistry, “Carol Davila” University of Medicine and Pharmacy, 020021 Bucharest, Romania; cristina.albu@umfcd.ro; 3 Department of Speciality Disciplines, “Titu Maiorescu” University, 031593 Bucharest, Romania; 4 Integrated Outpatient Department, “Prof. Dr. Agrippa Ionescu” Clinical Emergency Hospital, 011356 Bucharest, Romania; 5 Department of Internal Medicine, Family Medicine and Labor Medicine, Faculty of Medicine, “Carol Davila” University of Medicine and Pharmacy, 050474 Bucharest, Romania; lucia.bubulac@umfcd.ro; 6 Department of Complementary Science, Faculty of Medicine, “Carol Davila” University of Medicine and Pharmacy, 020021 Bucharest, Romania; raluca.gheorghe@umfcd.ro; 7 Department of Nephrology, Urology, Immunology and Immunology of Transplant, Dermatology, and Allergology, Faculty of Medicine, “Carol Davila” University of Medicine and Pharmacy, 020021 Bucharest, Romania; arsenie.spinu@umfcd.ro; 8 Faculty of Pharmacy, “Ovidius” University, 900470 Constanţa, Romania; emin.cadar@365.univ-ovidius.ro; 9 Department of Dental Medicine, Faculty of Medicine and Pharmacy, University of Oradea, 410073 Oradea, Romania; slavescudan@uoradea.ro; 10 Department of Orthodontics, Faculty of Dental Medicine, George Emil Palade University of Medicine, Pharmacy, Science, and Technology of Târgu Mureș, 540139 Târgu Mureș, Romania; mariana.pacurar@umfst.ro

**Keywords:** cytokines, chemokines, pathological root resorption, osteoclastogenesis, RANK/RANKL/OPG axis, biomarkers, gingival crevicular fluid, genetic polymorphisms, precision dentistry

## Abstract

Cytokines are key regulators of immune responses and tissue remodeling, playing a central role in physiological homeostasis and pathological inflammation. Dysregulation of cytokine signaling networks has been implicated in a wide range of diseases, where persistent inflammatory activation leads to progressive tissue destruction and impaired repair mechanisms. In the oral environment, cytokines critically influence the balance between tissue resorption and regeneration, particularly in processes involving dentin and alveolar bone remodeling. Pathological root resorption (PRR) represents a clinically significant model of cytokine-driven tissue destruction, characterized by the loss of dental hard tissues mediated by osteoclast-like cells within a dysregulated inflammatory microenvironment. Although mechanical, infectious, and iatrogenic factors are well-established triggers, they alone do not fully explain the variability in clinical outcomes, suggesting an important role for host-related factors. New research highlights the relationship between inflammatory signaling pathways, genetic susceptibility, and molecular biomarkers in shaping the onset and progression of PRR. In particular, the RANK/RANKL/OPG axis, cytokine networks, and gene polymorphisms have been identified as key determinants of osteoclast activation and resorptive activity. At the same time, advances in salivary and gingival crevicular fluid biomarker research provide new opportunities for early detection and real-time monitoring. Despite these advances, current knowledge remains fragmented, with heterogeneous study designs, inconsistent genetic associations, and a lack of standardized diagnostic protocols, all of which limit clinical translation. Therefore, a comprehensive and integrative synthesis of cytokine-mediated mechanisms in PRR is needed. This review aims to provide an updated and critical overview of cytokine and chemokine involvement in PRR, integrating molecular pathways, genetic determinants, and emerging biomarkers within a unified framework while highlighting translational implications for precision dentistry.

## 1. Introduction

Cytokines are key inflammatory mediators that coordinate immune responses and regulate tissue homeostasis across a wide spectrum of physiological and pathological conditions [1]. They play a central role in the pathogenesis of numerous diseases, including cancer, neurodegenerative disorders, autoimmune conditions, and chronic inflammatory diseases, by modulating cellular communication, immune activation, and tissue remodeling [2]. Dysregulation of cytokine signaling networks contributes to persistent inflammation and pathological tissue destruction, positioning cytokines as central mediators and clinically relevant biomarkers [3].

In the oral cavity, cytokines play a key role in regulating the balance between tissue repair and degradation, especially during bone and dentin remodeling [4]. Pathological root resorption (PRR) is a clinically significant example of cytokine-driven tissue destruction, in which inflammatory pathways act in concert with mechanical, microbial, and genetic factors to mediate tissue loss [5]. For consistency throughout this manuscript, the term “pathological root resorption (PRR)” is used to describe inflammatory root resorption processes affecting permanent teeth.

Root resorption represents a breakdown of dental hard tissues initiated by the activity of multinucleated odontoclasts [6]. While a normal phenomenon in deciduous teeth, its presence in permanent teeth signals a pathological process with potentially severe consequences [7]. Among the most recognized triggers are orthodontic forces, mechanical trauma, pulpal inflammation, and neoplastic growths [8,9]. Despite the commonality of these stimuli, not all individuals develop PRR, raising the question of intrinsic susceptibility [10]. Increasingly, evidence points to genetic predisposition as a major factor, with specific gene polymorphisms and molecular regulators contributing to individual variability in resorptive response [11].

The pathogenesis of PRR is conducted by a network of signaling molecules, inflammatory mediators, and genetic modulators [12]. The triad RANK/RANKL/OPG signaling governs the differentiation and survival of osteoclast-like cells [13,14]. Disruption in the balance of this axis has been implicated in exaggerated or prolonged root resorption. Additionally, growing evidence highlights the involvement of cytokines, matrix metalloproteinases, and epigenetic factors in this condition, with possible diagnostic and therapeutic implications [15].

This review aims to provide an integrated, up-to-date overview of the roles of cytokines and chemokines in the pathogenesis of PRR, with particular attention to their interactions with genetic susceptibility and emerging molecular biomarkers. It offers a new integrative perspective by simultaneously linking cytokine signaling, genetic susceptibility, and biomarker dynamics within a unified framework, an approach that remains insufficiently explored in the current literature. Unlike previous studies that address these components separately, this work brings together inflammatory signaling pathways, the RANK/RANKL/OPG axis, host genetic variability, and salivary and gingival crevicular fluid biomarkers into a unified conceptual framework. Furthermore, the review highlights the translational relevance of cytokine profiling for early diagnosis, risk stratification, and the development of personalized therapeutic strategies, while critically addressing current limitations and future research directions in this field.

Despite these advances, the recent literature has provided important insights into the biological and clinical aspects of PRR; however, most studies have approached the topic from relatively narrow or fragmented perspectives. For instance, recent systematic and narrative reviews have predominantly focused on clinical risk factors, orthodontic biomechanics, and general pathophysiology, with limited integration of molecular and genetic mechanisms [16]. Other contemporary works have explored the role of cytokines in orthodontic tooth movement and inflammation, emphasizing their regulatory effects on cellular activity, yet have not extended this analysis to a comprehensive genetic or biomarker-based framework [17]. Similarly, recent mechanistic reviews have examined signaling pathways involved in root resorption, such as osteoclastogenesis and inflammatory cascades, yet these studies often analyze molecular pathways in isolation, without linking them to host genetic variability or translational diagnostic strategies [18].

In contrast, the present review adopts a multidimensional integrative approach that simultaneously addresses cytokine signaling networks, genetic polymorphisms, epigenetic regulation, and fluid-based biomarkers within a unified conceptual framework. By linking these factors, this work moves beyond descriptive, single-axis analyses and offers a translational perspective aligned with precision dentistry. Specifically, it highlights how integrated molecular profiling may support early diagnosis, risk stratification, and the development of personalized therapeutic strategies in PRR, while also critically identifying current limitations and outlining future research directions.

Within this broader context, the role of cytokines extends far beyond localized inflammatory responses, constituting a shared molecular framework in diseases such as osteoporosis, rheumatoid arthritis, and cancer-related bone resorption, thereby underscoring their central role in both physiological bone remodeling and pathological tissue destruction. These shared molecular pathways highlight the convergence of cytokine-mediated mechanisms across local and systemic diseases, reinforcing the concept of a unified inflammatory framework underlying tissue resorption.

In this context, PRR may serve as a clinically accessible and biologically relevant model for studying cytokine-driven tissue destruction, offering insights applicable to broader systemic conditions characterized by dysregulated osteoclast activity and inflammatory signaling.

## 2. Review Methodology

### 2.1. Search Strategy and Selection Criteria

A comprehensive literature review was conducted to assess the current understanding of inflammatory, molecular, genetic, and biomarker-related mechanisms involved in PRR, with particular emphasis on cytokine signaling, osteoclastogenesis, genetic susceptibility, and emerging diagnostic biomarkers.

Electronic databases including PubMed/MEDLINE, Scopus, and Web of Science were systematically searched from database inception through January 2026. The search cutoff of January 2026 reflects the inclusion of articles published online ahead of print or in early 2026 within the database search window, consistent with the manuscript preparation timeline.

The search strategy incorporated Medical Subject Headings (MeSH) and free-text terms including “pathological root resorption”, “cytokines”, “chemokines”, “RANK/RANKL/OPG axis”, “osteoclastogenesis”, “biomarkers”, “gingival crevicular fluid”, “saliva”, “microRNAs”, “epigenetics”, “genetic polymorphisms”, and “precision dentistry”. Boolean operators (AND, OR) were applied to maximize sensitivity and specificity.

The initial search identified 1245 records. Following removal of duplicate records (*n* = 312), 933 articles underwent title and abstract screening, and 248 full-text articles were assessed for eligibility. Finally, 156 studies were included in the evidence synthesis.

The literature identification and evidence selection process is summarized in Supplementary Figure S1.

Only articles published in English were considered eligible. Inclusion criteria comprised original research articles, clinical investigations, biomarker studies, genetic association studies, in vitro studies, animal studies, and review papers that examined inflammatory pathways, cytokine-mediated mechanisms, biomarkers, or genetic determinants associated with PRR.

Exclusion criteria included studies focusing exclusively on non-inflammatory mechanisms, publications unrelated to cytokine-mediated pathways or molecular mechanisms in PRR, articles lacking sufficient methodological information, and studies with limited relevance to the objectives of this review.

Title/abstract screening and full-text eligibility assessment were independently performed by two investigators, and any disagreements were resolved through discussion and consensus. Additional relevant studies were identified through manual screening of reference lists from selected articles.

### 2.2. Quality Appraisal and Evidence Synthesis Framework

As this manuscript was designed as a critical integrative review rather than a formal systematic review or meta-analysis, the included literature was appraised using predefined qualitative criteria. Formal standardized instruments, such as the Cochrane Risk of Bias tool and the GRADE framework, were not applied because of the substantial heterogeneity of the available evidence, which comprised clinical observational studies, biomarker investigations, genetic association studies, animal models, in vitro experiments, and narrative or systematic reviews.

Rather than performing quantitative risk-of-bias scoring, the objective was to integrate diverse forms of evidence within a unified conceptual framework. Therefore, the reliability and relevance of the included literature were assessed according to predefined qualitative criteria, including direct relevance to PRR, clarity of the clinical or experimental model, adequacy of methodological and diagnostic descriptions, biological plausibility of the proposed mechanisms, reproducibility and consistency of findings across studies, sample size and population characteristics in clinical investigations, and translational relevance for diagnosis, risk stratification, or therapeutic application.

These criteria were used to critically interpret and prioritize the available evidence, distinguishing findings supported by stronger clinical data from preliminary mechanistic observations and exploratory experimental findings.

This approach facilitated the integration of heterogeneous data sources and supported a more comprehensive understanding of the interactions among cytokine signaling, genetic susceptibility, and biomarker dynamics in PRR.

### 2.3. Hierarchy of Evidence and Interpretation of Conflicting Findings

During evidence synthesis, a hierarchical interpretative framework was applied to account for differences in study design and translational relevance. Clinical studies and systematic reviews were assigned the highest interpretative weight, particularly when directly addressing PRR or clinically relevant inflammatory and biomarker-related outcomes. Genetic association studies and biomarker investigations in human populations were considered moderate-to-high level evidence because of their direct translational applicability, although findings were interpreted cautiously in view of population heterogeneity and methodological variability.

Animal studies were considered supportive mechanistic evidence, particularly for understanding inflammatory pathways and osteoclast regulation under controlled experimental conditions. In contrast, in vitro studies were primarily used to explain cellular and molecular mechanisms, including cytokine signaling pathways, osteoclastogenesis, and regulation of the RANK/RANKL/OPG axis. Findings derived exclusively from in vitro models were not considered sufficient to support direct clinical conclusions unless corroborated by corresponding human evidence.

When conflicting findings were identified across different study designs, greater interpretative weight was assigned to studies with direct clinical relevance, clearly defined diagnostic criteria, larger or better-characterized cohorts, and findings consistent with established biological mechanisms. Experimental observations that lacked validation in clinical studies were interpreted as preliminary evidence and discussed primarily within the context of future research directions.

### 2.4. Study Exclusion and Evidence Prioritization

As described in Section 2.1, studies presenting major methodological limitations were excluded during full-text eligibility assessment, consistent with the predefined exclusion criterion requiring adequate methodological information. Beyond these exclusions, no additional studies were removed solely on the basis of a formal quality scoring threshold. Studies with minor or moderate limitations that otherwise met the inclusion criteria were retained in the synthesis but assigned a restricted interpretative role, with their findings used primarily to contextualize existing gaps rather than to support primary mechanistic or clinical conclusions.

This approach enabled the inclusion of emerging or exploratory evidence while minimizing the influence of potentially less robust findings on the overall conclusions.

### 2.5. Critical Integrative Component of the Analysis

The critical integrative component of this review extended beyond a descriptive summary of published findings and involved an interpretative appraisal of the strength, consistency, and biological plausibility of the available evidence. Rather than presenting all studies with equivalent interpretative value, the synthesis considered differences in study design, methodological characteristics, and clinical applicability.

This interpretative framework directly influenced evidence synthesis by distinguishing findings supported by clinical investigations and reproducible observations from preliminary mechanistic evidence derived primarily from experimental models. Consequently, conclusions regarding cytokine-mediated pathways, genetic susceptibility, and biomarker applications were formulated according to the relative strength and translational relevance of the available evidence. Well-supported mechanisms and clinically reproducible findings were emphasized, whereas emerging biomarkers, experimental therapeutic concepts, and exploratory molecular associations were discussed more cautiously and primarily identified as areas requiring further validation and future investigation.

Accordingly, the purpose of this review was not only to summarize the literature but also to critically integrate diverse sources of evidence and provide an interpretative framework capable of supporting future research and potential clinical translation.

## 3. Cytokines and Chemokines in the Pathogenesis of PRR

Pathological root resorption is a multifactorial process characterized by the loss of mineralized dental tissues due to clastic activity, primarily resulting from a dysregulated inflammatory microenvironment [19]. In this environment, cytokines and chemokines serve as central mediators, integrating mechanical, infectious, and host-derived signals to coordinate biological responses that promote osteoclast differentiation and activation [20].

### 3.1. Cellular Sources and Inflammatory Microenvironment

The cytokine network implicated in PRR arises from both resident and infiltrating cells within the dentoalveolar complex [21]. Periodontal ligament fibroblasts, pulp cells, osteoblasts, cementoblasts, macrophages, and activated T lymphocytes all contribute to the local production of inflammatory mediators. The specific nature of the initiating stimulus significantly influences the resulting inflammatory response [22].

Depending on the type of trigger, inflammatory signaling in PRR may be initiated by mechanical stimuli or by microbial factors, leading to distinct but converging inflammatory pathways that ultimately promote a pro-resorptive microenvironment [23].

#### Sterile Versus Infection-Driven Inflammation in PRR

Pathological root resorption can result from either sterile or infection-driven inflammation, which share some mechanisms but also exhibit distinct differences [24]. Sterile inflammation, typically induced by orthodontic forces or trauma, involves the release of damage-associated molecular patterns (DAMPs) from stressed or injured cells [25]. These molecules activate local cells within the periodontal ligament and adjacent tissues, initiating pro-inflammatory cytokine production via mechanotransduction, even in the absence of microbial presence [26]. In contrast, infection-driven inflammation arises when immune cells recognize microbial components, leading to their activation and the stimulation of distinct cytokine pathways. While sterile inflammation is generally transient and localized, prolonged mechanical stress can shift this response toward pathological resorption [27].

Infection-driven inflammation is initiated by microbial invasion, as observed in conditions such as pulpal necrosis or apical periodontitis [28]. In these cases, bacterial pathogen-associated molecular patterns (PAMPs) activate innate immune receptors, primarily Toll-like receptors, resulting in a more robust and sustained inflammatory response [29]. This process leads to increased production of pro-inflammatory cytokines, enhanced concentration of immune cells, and pronounced stimulation of osteoclastogenesis [30]. Compared to sterile inflammation, infection-driven responses are more intense, persist longer, and pose a higher risk of progressive, clinically significant root resorption [5].

Despite differences in their initiating signals and overall magnitude, both inflammatory pathways ultimately converge through cytokine-mediated activation of the RANK/RANKL/OPG axis, which promotes osteoclast differentiation and subsequent mineralized tissue degradation [13,31].

### 3.2. Pro-Inflammatory Cytokines and Osteoclast Activation

Pro-inflammatory cytokines are key mediators in PRR, significantly promoting clastic cell activity. Interleukin-1 beta (IL-1β), interleukin-6 (IL-6), and tumor necrosis factor-alpha (TNF-α) are frequently linked to increased osteoclastogenesis [32,33].

IL-1β amplifies the resorptive process by inducing the expression of osteoclastogenic factors in stromal and immune cells, as well as by extending the survival and activity of mature osteoclasts [34]. IL-6 perpetuates the inflammatory cascade and facilitates the differentiation of osteoclast precursors, particularly in chronic inflammatory states [35]. TNF-α collaborates with other cytokines to promote the recruitment and differentiation of osteoclast precursors and, under certain conditions, can directly induce osteoclastogenesis [36].

These cytokines function within an interconnected signaling network that sustains and amplifies local inflammation, thereby promoting persistent osteoclastic resorptive activity [37].

### 3.3. Cytokine Balance and Intracellular Signaling Pathways in PRR

Pro-inflammatory cytokines: IL-1β, IL-6, and TNF-α are primary drivers of the inflammatory cascade in PRR; however, disease progression depends on the dynamic balance between pro- and anti-inflammatory mediators. Anti-inflammatory cytokines, including interleukin-10 (IL-10) and transforming growth factor-beta (TGF-β), serve as key regulators that limit excessive inflammation and modulate tissue remodeling. IL-10 inhibits the production of pro-inflammatory cytokines and suppresses osteoclast differentiation, whereas TGF-β promotes tissue repair and may exert context-dependent effects on mineralized tissue resorption [38].

Disruption of cytokine equilibrium, marked by predominant pro-inflammatory signaling over regulatory mechanisms, represents a critical event in the transition from physiological remodeling to pathological resorption. Therefore, PRR should be considered not only a consequence of increased pro-inflammatory activity but also a manifestation of insufficient anti-inflammatory regulation [39].

At the intracellular level, cytokine-mediated responses in PRR involve multiple interconnected signaling pathways that regulate osteoclast differentiation, activation, and survival. Among these pathways, nuclear factor kappa B (NF-κB), mitogen-activated protein kinase (MAPK), and Janus kinase/signal transducer and activator of transcription (JAK/STAT) signaling pathways are considered central regulators of osteoclastic activity [20,40].

Activation of the NF-κB pathway is initiated following RANKL binding to RANK on osteoclast precursor cells. This interaction recruits adaptor proteins, particularly TNF receptor-associated factor 6 (TRAF6), which subsequently activate the IκB kinase complex. Phosphorylation and degradation of IκB proteins permit nuclear translocation of NF-κB transcription factors, leading to increased expression of genes involved in osteoclast differentiation and function, including NFATc1, cathepsin K, and TRAP [13,38,40,41].

MAPK pathways, including ERK, JNK, and p38, also participate in osteoclastogenesis by regulating transcription factors involved in cell proliferation, differentiation, and survival [40]. Activation of MAPK signaling contributes to amplification of inflammatory responses and enhances expression of genes associated with resorptive activity [42].

In parallel, cytokines such as IL-6 activate the JAK/STAT signaling pathway through receptor-associated Janus kinases. Activated STAT proteins translocate into the nucleus and regulate genes involved in inflammatory amplification, osteoclast precursor maturation, and immune responses [43,44,45]. Crosstalk among NF-κB, MAPK, and JAK/STAT signaling pathways establishes positive feedback mechanisms that sustain osteoclast activation and contribute to persistent tissue resorption [20,40].

Collectively, these intracellular signaling networks represent potential therapeutic targets because modulation of these pathways may attenuate inflammatory amplification and pathological osteoclastic activity [46,47].

**Figure 1:**
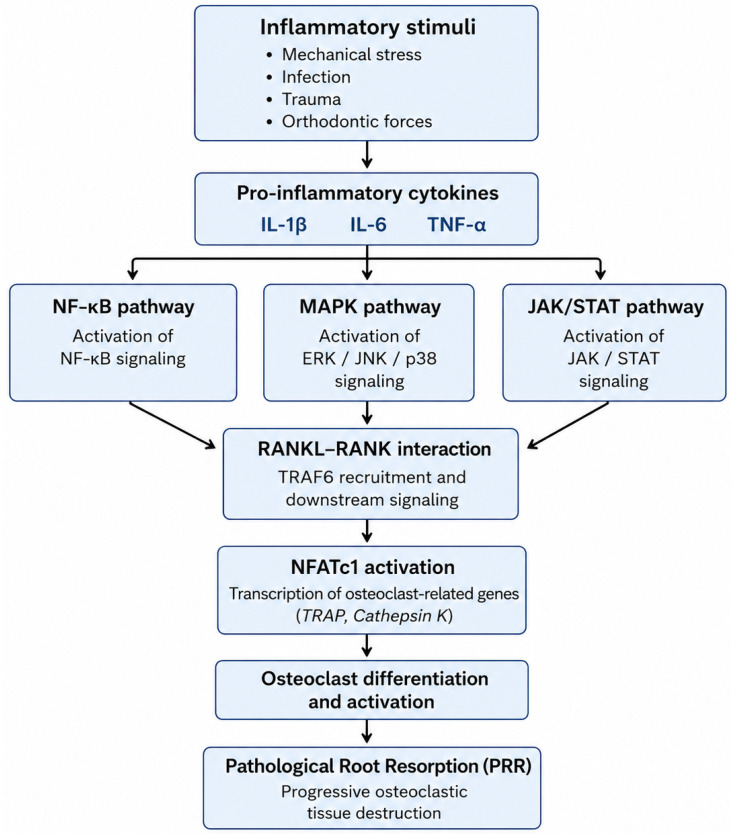
Integrated signaling pathways involved in pathological root resorption (PRR). Inflammatory stimuli, including mechanical stress, infection, trauma, and orthodontic forces, induce the release of pro-inflammatory cytokines such as IL-1β, IL-6, and TNF-α. These mediators activate RANKL–RANK signaling and TRAF6-dependent downstream pathways, including NF-κB, MAPK, and JAK/STAT cascades. Convergent signaling promotes NFATc1-mediated transcription of osteoclast-related genes, including TRAP and cathepsin K, leading to osteoclast differentiation, activation, and progressive pathological tissue resorption. Abbreviations: PRR—Pathological Root Resorption; IL-1β—Interleukin-1 beta; IL-6—Interleukin-6; TNF-α—Tumor Necrosis Factor alpha; RANKL—Receptor Activator of Nuclear Factor Kappa-B Ligand; RANK—Receptor Activator of Nuclear Factor Kappa-B; TRAF6—TNF Receptor-Associated Factor 6; NF-κB—Nuclear Factor Kappa-B; MAPK—Mitogen-Activated Protein Kinase; ERK/JNK/p38—Extracellular Signal-Regulated Kinase/JNK—c-Jun N-terminal Kinase/p38—p38 Mitogen-Activated Protein Kinase; JAK/STAT—Janus Kinase/Signal Transducer and Activator of Transcription; NFATc1—Nuclear Factor of Activated T Cells Cytoplasmic 1; TRAP—Tartrate-Resistant Acid Phosphatase.

The principal cytokine-mediated intracellular signaling interactions involved in osteoclast activation and progression of PRR are summarized schematically in Figure 1.

### 3.4. The RANK/RANKL/OPG Axis in PRR

The RANK/RANKL/OPG signaling pathway is a central mechanism connecting inflammation to tissue resorption [40]. RANKL, produced by periodontal ligament cells, osteoblast-lineage cells, and activated immune cells, binds to the RANK receptor on osteoclast precursors, thereby initiating their differentiation into active osteoclasts [48,49]. In contrast, osteoprotegerin (OPG) acts as a decoy receptor that inhibits the RANKL-RANK interaction [50].

In PRR, inflammatory cytokines induce increased RANKL expression and reduced OPG availability, resulting in an elevated RANKL/OPG ratio [51,52]. This imbalance is a key factor that drives enhanced osteoclast activity and progressive tissue resorption [53]. Regulation of this signaling axis in dental tissues may differ from systemic bone remodeling, highlighting the need for tissue-specific investigation [54].

### 3.5. Chemokines and Recruitment of Osteoclast Precursors

Chemokines are critical in sustaining PRR by regulating the migration and accumulation of immune and progenitor cells at sites of inflammation. CXCL8 (interleukin-8) mediates the early recruitment of neutrophils and has been implicated in promoting osteoclast differentiation under inflammatory conditions [55]. CCL2 (monocyte chemoattractant protein-1) facilitates the recruitment of monocytes and macrophages, which act as precursors for osteoclasts [56].

These mechanisms enable chemokines to expand the population of cells capable of differentiating into clastic cells, thereby reinforcing the resorptive process [57]. Additionally, chemokine signaling contributes to persistent inflammation, resulting in a self-sustaining inflammatory microenvironment [58].

### 3.6. Matrix Degradation and Cytokine–MMP Interactions

The resorptive process is further supported by the activation of matrix metalloproteinases (MMPs), particularly MMP-9, which degrade extracellular matrix components and facilitate access of clastic cells to mineralized tissues [59]. The expression and activity of MMPs are regulated by pro-inflammatory cytokines, establishing a functional link between inflammatory signaling and structural tissue breakdown [15].

This coordinated interaction between cytokines and matrix-degrading enzymes underscores the dual inflammatory and degradative nature of PRR.

### 3.7. From Physiological Remodeling to Pathological Resorption

Root resorption is a physiological process during tooth exfoliation; however, PRR represents a pathological deviation marked by excessive and sustained cytokine signaling [60]. Under physiological conditions, inflammatory mediators are tightly regulated and balanced by inhibitory mechanisms [12,61]. In contrast, PRR is characterized by prolonged cytokine expression, disruption of regulatory feedback, and an imbalance that favors osteoclast activation, ultimately resulting in irreversible tissue loss [42].

### 3.8. Translational Relevance

The characterization of cytokine and chemokine networks in PRR has important translational implications [62]. Elevated levels of key mediators in gingival crevicular fluid or saliva may serve as early indicators of active resorption [63]. Furthermore, modulation of cytokine pathways represents a potential therapeutic strategy, although current evidence remains limited and largely experimental [46]. The clinical application of targeted anti-cytokine approaches requires further validation in well-designed studies [47].

Despite the growing body of evidence supporting the role of cytokines and related molecular pathways in PRR, the current understanding remains partially constrained by several methodological and translational challenges [18]. Much of the available data is derived from heterogeneous experimental models and relatively small clinical cohorts, which may not fully reflect the complexity of human disease. In addition, reported associations between cytokine gene polymorphisms and PRR susceptibility are often inconsistent across populations, highlighting the influence of genetic background and environmental variability [64]. These limitations highlight the need for more powerful, standardized, and translational research, as further discussed in Section 8.

## 4. Genetic Regulation of Cytokine-Mediated Responses in PRR

Susceptibility to PRR is influenced by factors beyond environmental or mechanical stimuli. Recent evidence emphasizes the significant role of host genetics in modulating the onset, severity, and progression of resorptive processes [65]. An expanding body of research has identified various genetic markers that affect individual responses to inflammatory triggers, mechanical forces, and tissue injury [66]. These genetic variations, frequently manifesting as single–nucleotide polymorphisms (SNPs), can alter the expression and functional activity of regulatory molecules involved in osteoclastogenesis, immune signaling, and dentin integrity [67]. Explaining these genetic determinants clarifies the molecular basis of PRR and facilitates the development of predictive diagnostics and personalized therapies [68]. The following subsections review the principal gene families and polymorphisms currently associated with PRR susceptibility.

### 4.1. Cytokine Gene Polymorphisms

**Figure 2:**
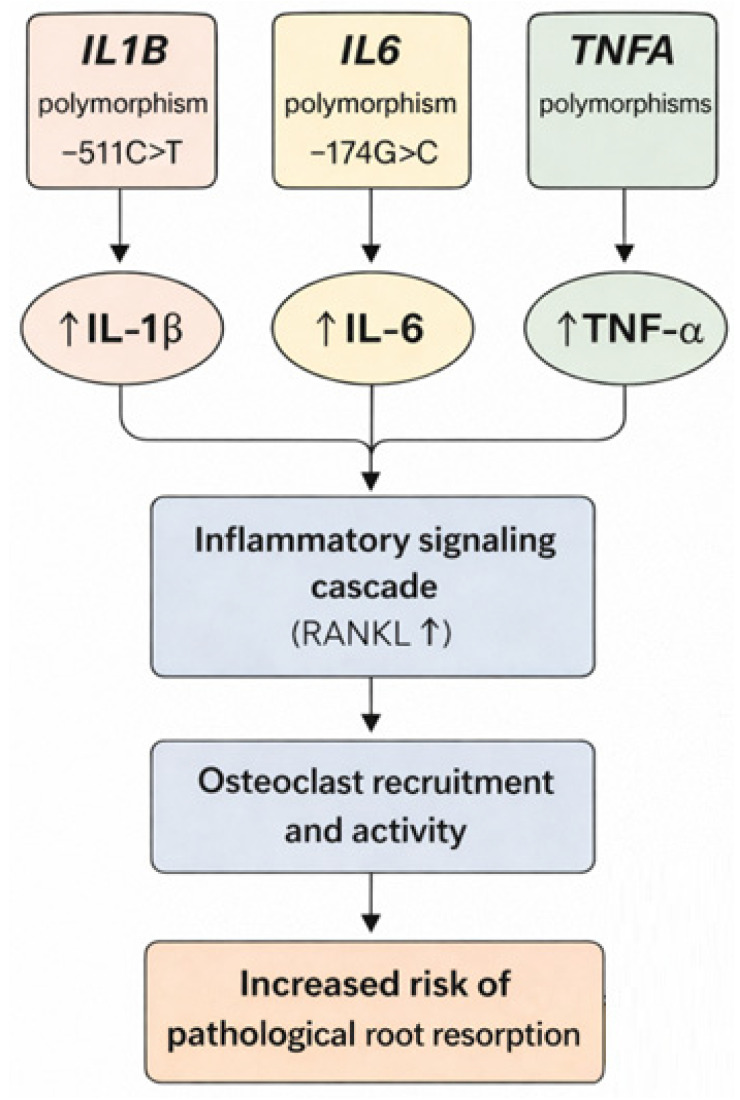
Representative cytokine-related genetic determinants involved in PRR. Polymorphisms in cytokine genes (IL1B, IL6, TNFA) are associated with increased expression of pro-inflammatory cytokines (IL-1β, IL-6, TNF-α). These alterations enhance inflammatory signaling pathways, promote RANKL-mediated osteoclastogenesis, and increase osteoclast recruitment and activity, ultimately contributing to PRR.

Inflammatory cytokines are central to the cellular signaling networks that activate and sustain osteoclastic activity during PRR. Among them, interleukin-1 beta (IL-1β), interleukin-6 (IL-6), and tumor necrosis factor-alpha (TNF-α) are especially important due to their pro-resorptive roles [69]. Genetic polymorphisms within the genes encoding these cytokines—namely IL1B, IL6, and TNFA—have been shown to influence their expression and activity levels. For example, the IL1B −511C>T polymorphism has been associated with increased transcriptional activity and elevated IL-1β concentrations in gingival crevicular fluid (GCF), a biomarker-rich medium routinely analyzed in periodontal and orthodontic research [70,71]. During orthodontic tooth movement, this elevation correlates with accelerated osteoclast recruitment and activity, leading to an increased risk of PRR [72]. Similarly, IL-6 gene variants, such as −174G>C, are linked to greater systemic and local cytokine levels, which can potentiate the inflammatory cascade in the periodontal ligament [73]. TNF-α, encoded by the TNFA gene, exerts a synergistic effect by amplifying inflammatory signaling and promoting a pro-resorptive cellular environment [71,74]. These cytokine gene polymorphisms contribute to an individualized inflammatory response threshold that may explain why some patients develop significant resorption under similar mechanical or infectious stresses while others do not (Figure 2).

### 4.2. RANK/RANKL/OPG Axis

The RANK/RANKL/OPG signaling axis is the central molecular switch that controls osteoclast differentiation, activation, and survival. RANKL (Receptor Activator of Nuclear factor Kappa-B Ligand), encoded by the TNFSF11 gene, binds to its receptor RANK on pre-osteoclasts, triggering their maturation into bone-resorbing cells [75]. Osteoprotegerin (OPG), encoded by TNFRSF11B, functions as a soluble decoy receptor that binds RANKL and prevents its interaction with RANK. The balance between RANKL and OPG is thus critical in maintaining bone and root homeostasis. Genetic polymorphisms in TNFSF11 can lead to increased RANKL expression, tilting the balance toward enhanced osteoclastogenesis [76,77,78]. Conversely, variants in TNFRSF11B that reduce OPG production or function may exacerbate this imbalance, allowing unchecked resorptive activity [79]. In individuals undergoing orthodontic treatment, a genetic predisposition may significantly elevate their risk of developing PRR, even under otherwise controlled biomechanical conditions [80]. Moreover, these polymorphisms may have cumulative or synergistic effects when combined with pro-inflammatory cytokine gene variants, supporting the concept of PRR as a polygenic and multifactorial condition [81].

### 4.3. Vitamin D Receptor (VDR)

The Vitamin D receptor (VDR) exercises immunomodulatory and mineral-regulating effects in periodontal tissues by modulating osteoclastic activity through calcium-phosphorus homeostasis and inflammatory signaling pathways [82]. Polymorphisms in the VDR gene, such as FokI (rs2228570), TaqI (rs731236), and BsmI (rs1544410), have been associated with changes in receptor function and expression [83]. These genetic variations influence the transcriptional regulation of downstream inflammatory and bone-remodeling pathways [84]. The FokI variant, for instance, produces a shorter and more transcriptionally active receptor, which may enhance pro-resorptive gene expression following vitamin D stimulation [85]. Among orthodontic patients, the presence of risk alleles is linked to increased inflammatory responses and reduced protective signaling, thereby elevating the risk of PRR [86]. Furthermore, vitamin D deficiency may interact with VDR polymorphisms, further compromising bone metabolism and immune function, highlighting a gene-environment interaction with significant implications for risk assessment and treatment planning in dental practice [87].

### 4.4. P2RX7 and DSPP

Beyond the classical cytokine and bone metabolism genes, emerging evidence points to the involvement of additional genetic regulators such as P2RX7 and DSPP. The P2RX7 gene encodes a purinergic receptor involved in cellular apoptosis and cytokine release, particularly in osteoclasts and macrophages [88]. Loss-of-function polymorphisms in this gene may impair apoptosis, resulting in prolonged osteoclast survival and sustained resorptive activity [89]. This mechanism may create a cellular environment that favors sustained root resorption in response to inflammatory or mechanical stimuli. In parallel, DSPP (Dentin Sialophosphoprotein), a critical gene in dentin matrix formation, has been associated with internal resorption and structural tooth abnormalities [90]. Mutations in DSPP compromise the integrity of dentin and alter the adhesion dynamics between dentin and odontoclasts, promoting the attachment and resorptive behavior [91]. Proteolytic fragments of DSPP released during stress or inflammation may further stimulate RANKL expression in dental pulp cells, perpetuating the resorptive cycle [92].

Together, P2RX7 and DSPP highlight the complexity of PRR genetics, illustrating the contribution of bone-regulating and dentin-structuring pathways to disease progression. A synthesis of the key genetic determinants involved in cytokine-mediated pathways in PRR, including their functional and clinical implications, is provided in Table 1.

These genetic determinants support the concept of PRR as a polygenic and multifactorial condition, with direct implications for risk stratification and personalized treatment planning.

Despite the growing body of evidence supporting the role of genetic polymorphisms in PRR, the current literature presents several important limitations that must be critically considered. One of the major challenges is the inconsistency of reported associations across different populations. Variations in allele frequency, genetic background, environmental exposures, and study design contribute to heterogeneous findings, particularly for cytokine-related genes such as IL1B, IL6, and TNFA [93]. As a result, genetic markers identified in one population may not be directly generalizable to others.

Furthermore, the strength of the reported associations is often modest, with most polymorphisms exerting small to moderate effects on disease susceptibility rather than acting as independent predictive factors [94]. This highlights the polygenic and multifactorial nature of PRR, in which individual genetic variants contribute cumulatively rather than deterministically to disease risk.

From a clinical perspective, the applicability of these genetic findings remains limited and not yet suitable for routine clinical implementation [95]. Although several candidate genes have been proposed as potential risk markers, their integration into routine clinical practice is currently constrained by the lack of standardized testing protocols, limited validation in large-scale studies, and insufficient evidence regarding cost-effectiveness. Therefore, while genetic profiling holds promise for future risk stratification and personalized treatment planning, its use in clinical decision-making remains largely theoretical at present.

## 5. Emerging Diagnostic Biomarkers

One of the foremost challenges in managing PRR is the inability to detect early molecular changes before irreversible structural damage occurs [96]. Conventional imaging modalities, such as radiographs and cone-beam computed tomography (CBCT), have limited sensitivity in the early stages of resorption, by which time substantial dentin or cementum loss may already be present [97]. This diagnostic limitation has driven growing interest in identifying molecular biomarkers detectable by non-invasive approaches.

The main cytokine-related and molecular biomarkers associated with PRR, together with their biological roles and diagnostic potential, are summarized in Table 2.

To facilitate clinical interpretation, biomarkers can be stratified by clinical readiness, ranging from well-supported candidates with translational potential to emerging experimental markers requiring further validation.

Incorporating these biomarkers into multi-parameter diagnostic panels could enhance early detection and facilitate real-time monitoring of resorptive activity. Several cytokines, including IL-1β, IL-6, and TNF-α, serve as both central mediators of osteoclastogenesis and clinically relevant diagnostic indicators.

Gingival crevicular fluid (GCF) and saliva are considered optimal diagnostic media because of their accessibility, representative biochemical profiles, and responsiveness to local inflammatory and resorptive changes [98]. Elevated matrix metalloproteinase-9 (MMP-9) levels, an enzyme involved in dentin and collagen degradation, are consistently linked to active resorption, especially during orthodontic tooth movement [99]. Soluble RANKL (sRANKL) is also proposed as a surrogate marker of osteoclastic activity, with the sRANKL-to-OPG ratio correlating with the degree of root surface loss [100].

MicroRNAs (miRNAs), which are small non-coding RNAs regulating gene expression post-transcriptionally, constitute a promising class of biomarkers. Specific miRNAs, including miR-29b and miR-21, are elevated in patients undergoing orthodontic treatment who exhibit resorptive activity [101]. These molecules not only indicate ongoing cellular processes but may also contribute to disease progression by increasing RANKL expression and MMP-9 secretion, functioning as both biomarkers and mediators of PRR [102].

Developing point-of-care diagnostic tools that detect these biomarkers in real time could transform clinical management. Such approaches would facilitate early identification of high-risk individuals, timely interventions, and personalized therapeutic adjustments. Consequently, biomarker-based diagnostics have the potential to enhance clinical decision-making.

From a translational standpoint, distinguishing between experimental biomarkers and those with clinical applicability is essential. Although many cytokines, matrix metalloproteinases, and microRNAs have been proposed as biomarkers for PRR, only a few have been consistently validated in clinical studies [103]. Biomarkers such as IL-1β, IL-6, TNF-α, MMP-9, and the RANKL/OPG ratio demonstrate the strongest evidence, because of their reproducible association with inflammatory activity and disease progression [104,105]. Conversely, emerging biomarkers, including specific microRNAs and less-studied cytokines, remain experimental and require further validation in large-scale clinical studies.

The diagnostic performance of these biomarkers is another critical consideration. While multiple studies indicate that elevated cytokine and MMP levels correlate with active resorption, data on sensitivity, specificity, and predictive value are limited and heterogeneous [106]. Variability in sampling techniques, analytical methods, and patient populations further complicates direct comparisons across studies.

Additionally, the absence of standardized protocols for biomarker collection, processing, and quantification is a significant barrier to clinical implementation [107]. Differences in gingival crevicular fluid sampling, saliva collection, and laboratory assays substantially impact biomarker reliability and reproducibility. As a result, despite their biological relevance, most biomarkers are not yet appropriate for routine clinical application.

Overall, while biomarker-based approaches offer significant potential for early detection and longitudinal monitoring of PRR, additional well-designed clinical studies are necessary to establish standardized methodologies, validate diagnostic accuracy, and determine clinically relevant thresholds.

## 6. Epigenetics and Gene–Environment Interactions

Epigenetic regulation is essential for modulating cytokine expression in PRR. Genetic polymorphisms establish individual susceptibility, whereas epigenetic modifications offer a dynamic regulatory layer that integrates genetic predisposition with environmental influences. Mechanisms including DNA methylation, histone acetylation, and chromatin remodeling govern gene locus accessibility to transcriptional machinery without altering the DNA sequence [108]. These reversible changes are highly responsive to lifestyle and environmental factors, which are critical for explaining variability in PRR outcomes among genetically similar individuals.

Within PRR, hypermethylation of the OPG gene promoter has been identified in trauma-induced resorption, which may suppress OPG expression and shift the RANKL/OPG balance toward resorption [109]. Additionally, histone modification patterns in the promoters of IL-1β and TNF-α modulate their responsiveness to mechanical stress or microbial infection [110]. These epigenetic alterations act as molecular switches that determine whether a transient inflammatory event advances to pathological resorption [111].

Interactions between cytokines and epigenetic regulators are bidirectional and may substantially influence the progression of PRR. Pro-inflammatory cytokines such as IL-1β, IL-6, and TNF-α may contribute to epigenetic alterations through regulation of DNA methyltransferases, histone-modifying enzymes, and chromatin-remodeling complexes [112]. Conversely, epigenetic modifications can regulate cytokine gene expression by altering chromatin accessibility and transcriptional activity. For example, changes in histone acetylation patterns within promoter regions of inflammatory genes may enhance transcription of cytokines associated with osteoclast activation, whereas DNA methylation changes may suppress protective regulatory pathways [113]. These interactions may establish regulatory feedback mechanisms capable of sustaining inflammatory signaling and promoting prolonged osteoclastic activity in PRR. In particular, DNMT3a-mediated DNA methylation of the IRF8 locus—a key negative regulator of osteoclastogenesis—has been identified as a critical epigenetic mechanism that stabilizes osteoclast differentiation by suppressing anti-resorptive gene expression [114,115]. Collectively, these bidirectional interactions suggest that cytokines and epigenetic regulators function within interconnected molecular networks capable of amplifying inflammatory responses and influencing susceptibility to PRR.

Environmental factors, including vitamin D deficiency, inadequate nutrition, smoking, and chronic low-grade inflammation, interact with epigenetic regulators [116]. For example, insufficient vitamin D impairs calcium metabolism and may enhance pro-resorptive gene expression through hypomethylated vitamin D receptor-related pathways [117]. Likewise, high-sugar or pro-inflammatory diets elevate systemic cytokine levels, amplifying the effects of genetic variants in IL1B or TNFA [118].

**Figure 3:**
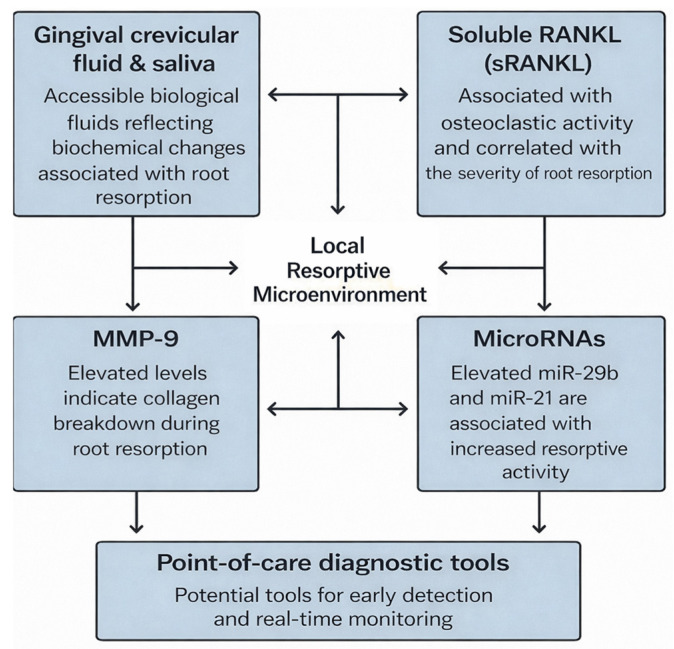
Biomarker-based detection and monitoring of PRR. Gingival crevicular fluid and saliva represent accessible biological fluids that reflect biochemical changes associated with PRR. Key biomarkers include matrix metalloproteinase-9 (MMP-9), soluble RANKL (sRANKL), and regulatory microRNAs such as miR-29b and miR-21, which are associated with osteoclastic activity and tissue remodeling. These molecular indicators interact within the local resorptive microenvironment and correlate with the severity of root resorption. Their combined assessment enables early detection of subclinical resorptive processes and supports real-time monitoring through point-of-care diagnostic tools.

Comprehending gene, environment, and epigenetic interactions is essential for developing comprehensive risk models. These models should integrate static genetic data with dynamic biomarkers and modifiable lifestyle factors, facilitating preventive and therapeutic strategies (Figure 3).

Beyond classical epigenetic mechanisms, recent evidence demonstrates that microRNA-mediated regulatory networks modulate cytokine expression and osteoclast activity in PRR. MicroRNAs, including miR-21 and miR-29b, post-transcriptionally regulate key genes involved in RANKL signaling, matrix degradation, and inflammatory pathways [119,120]. These molecules function within complex and interconnected regulatory networks, influencing both pro-inflammatory and anti-inflammatory signaling cascades and contributing to the balance between tissue resorption and repair [121].

Mechanistically, microRNAs regulate PRR through post-transcriptional modulation of target messenger RNAs involved in inflammatory signaling and osteoclast differentiation. miR-21 has been associated with enhanced osteoclastogenesis through suppression of programmed cell death protein 4 (PDCD4), thereby facilitating RANKL-mediated signaling and activation of downstream pathways involved in clastic cell differentiation [122,123]. In contrast, members of the miR-29 family have been implicated in extracellular matrix remodeling and bone metabolism through regulation of genes associated with collagen synthesis and Wnt signaling pathways [124]. Emerging evidence also suggests regulatory roles for miR-146a and miR-155 in inflammatory feedback mechanisms through modulation of NF-κB activity and cytokine expression, with miR-155 having been reported to target SOCS1 and modulate osteoclast differentiation during orthodontic tooth movement, and miR-146a has been reported to modulate TRAF6/NF-κB signaling to regulate osteoclastic bone resorption [125,126]. Through these interconnected regulatory networks, microRNAs may influence the balance between inflammatory activation, tissue remodeling, and reparative responses in PRR.

Epigenetic regulation constitutes a promising therapeutic target for modulating disease progression. Potential strategies encompass histone deacetylase (HDAC) inhibitors, DNA methylation modulators, and microRNA-based therapies aimed at restoring regulatory balance within the inflammatory microenvironment [127]. While these approaches remain largely experimental in PRR, they have demonstrated encouraging results in other inflammatory and bone-related conditions, supporting their potential translational relevance [128,129].

Integrating epigenetic profiling into precision medicine frameworks may yield a more comprehensive understanding of individual disease susceptibility and treatment response. Combining genetic, epigenetic, and biomarker data could facilitate the development of personalized risk models and targeted therapeutic strategies for individual patients. In this context, epigenetic markers and microRNA signatures may serve as diagnostic tools and therapeutic targets, thereby bridging the gap between molecular research and clinical application.

## 7. Personalized Therapeutics and Clinical Translation

The confluence of genomic science and dental practice is ushering in a new era of personalized therapeutics for PRR management [130]. Rather than relying on generalized treatment protocols, clinicians can now tailor interventions to an individual’s genetic and molecular profile. This approach begins with risk stratification using validated genetic markers—such as polymorphisms in RANKL, OPG, IL-1β, and VDR—which can identify patients who are more likely to exhibit aggressive resorptive responses.

For high-risk individuals, orthodontic treatment plans may be modified to apply lighter forces, implement extended rest intervals, or shorten total treatment duration [131,132]. In certain cases, pharmacological adjuncts may be considered, including bisphosphonates, which inhibit osteoclast function, or monoclonal antibodies targeting RANKL, such as denosumab [133]. These agents, though traditionally used in systemic bone disorders, may have potential for localized dental applications to manage severe or refractory PRR [134].

Salivary and GCF biomarker monitoring can further enhance therapeutic precision by providing real-time feedback on the resorptive status [135]. Elevated MMP-9 or miRNA levels during treatment could prompt immediate modifications in force application or trigger prophylactic interventions, thus minimizing irreversible damage [136].

**Figure 4:**
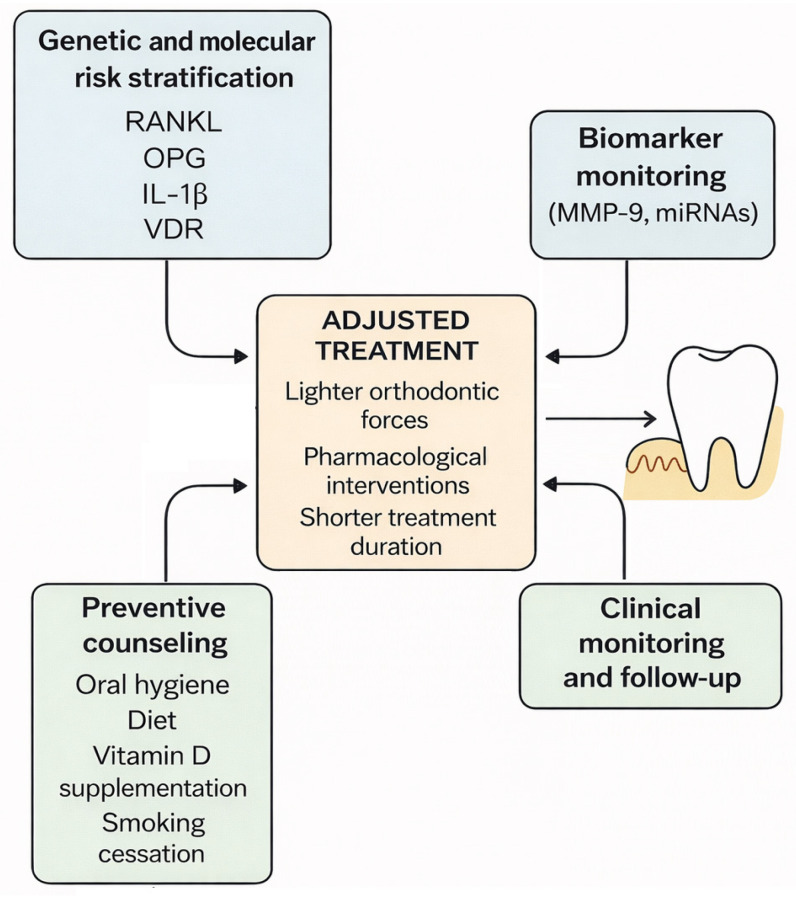
Personalized therapeutic strategies for PRR based on genetic and molecular profiling. The integration of genetic and molecular profiling enables individualized management of PRR. Risk stratification based on genetic markers (e.g., RANKL, OPG, IL-1β, VDR) is combined with biomarker monitoring (e.g., MMP-9, microRNAs) to guide clinical decision-making. Personalized interventions may include adjustment of orthodontic forces, pharmacological approaches targeting osteoclast activity, preventive strategies such as optimization of oral hygiene and vitamin D status, and continuous clinical monitoring to improve treatment outcomes.

Importantly, personalized care also encompasses preventive counseling. Patients with genetic or molecular predispositions may benefit from enhanced oral hygiene regimens, anti-inflammatory diets, vitamin D supplementation, or cessation programs for tobacco use—all of which can modulate risk trajectories [137]. As more evidence accumulates, standardized clinical guidelines will be necessary to integrate genetic screening [138,139] and molecular diagnostics into mainstream orthodontic and endodontic protocols (Figure 4).

The cytokine-driven mechanisms observed in PRR share significant similarities with those involved in systemic inflammatory diseases such as rheumatoid arthritis, osteoporosis, and cancer-related bone resorption. This underscores the translational relevance of PRR as a model for cytokine-mediated tissue destruction and supports its integration into the broader framework of inflammatory and precision medicine.

Experimental concepts in personalized therapeutics for PRR primarily focus on targeted modulation of inflammatory and osteoclastic pathways. Potential approaches include anti-cytokine therapies, inhibition of RANKL-mediated signaling, epigenetic modulators, and microRNA-based interventions [1,40]. Although anti-resorptive agents such as bisphosphonates and anti-RANKL therapies have demonstrated efficacy in systemic bone disorders, their application in PRR remains largely experimental and has not been validated for routine dental use [133,134]. Notably, a recent animal study demonstrated that RANKL-expressing senescent cells induced by mechanical stress play a pivotal role in odontoclastogenesis and apical root resorption, and that oral administration of the senolytic cocktail dasatinib and quercetin markedly reduced these senescent cells and alleviated root resorption, identifying a novel therapeutic target directly relevant to PRR [140]. Localized delivery systems capable of selectively modulating inflammatory pathways may represent a promising future strategy while minimizing systemic adverse effects. In this regard, NF-κB decoy oligodeoxynucleotide-loaded PLGA nanospheres administered locally during orthodontic tooth movement have been shown to suppress osteoclastogenesis, downregulate TNF-α, IL-1β, and RANKL expression, and attenuate alveolar bone resorption in preclinical models, supporting the translational potential of localized nanomedicine approaches for dental applications [141].

Experimentally, local OPG gene transfer to periodontal tissue has been shown to inhibit RANKL-mediated osteoclastogenesis and attenuate experimental tooth movement in animal models, demonstrating the potential of site-specific anti-resorptive interventions targeting the RANKL/OPG axis [142]. More recently, the IL-1 receptor antagonist anakinra has been shown to inhibit IL-1β-mediated osteoclast formation by periodontal ligament fibroblasts in vitro, supporting the translational relevance of anti-cytokine strategies for periodontal inflammatory bone loss [143]. MicroRNA-based therapeutic strategies have also been explored: miR-29b has been shown to negatively regulate human osteoclastic cell differentiation and function through downregulation of SP1 and NFATc1, impairing osteoclast activity even in the presence of robust RANKL stimulation [144]; building on this, localized nanoparticle-mediated delivery of miR-29b has demonstrated capacity to normalize bone homeostasis dysregulation, positioning miRNA-based nanomedicine as a candidate platform for site-specific anti-resorptive intervention [145]. Furthermore, the pan-HDAC inhibitor vorinostat (SAHA) has demonstrated capacity to suppress RANKL-induced NFATc1 activity, disrupt osteoclastic bone resorption in vitro, and attenuate ovariectomy-induced bone loss in vivo, supporting the translational potential of epigenetic modulators as adjunct anti-resorptive therapies [146].

From a clinical perspective, biomarker-guided approaches currently remain limited primarily to risk assessment and monitoring applications rather than direct therapeutic decision-making. Molecular profiling using cytokine patterns, genetic susceptibility markers, and salivary or gingival crevicular fluid biomarkers may assist in identifying patients at increased risk for PRR [135]. However, substantial challenges remain, including lack of standardized thresholds, variability among patient populations, and insufficient prospective validation studies [63]. Therefore, current applications should be regarded as supportive tools for clinical assessment rather than established therapeutic decision-making systems.

## 8. Ethical and Practical Considerations

The integration of genetic and molecular tools into clinical dentistry, while promising, raises critical ethical and practical concerns that must be carefully addressed [147]. At the forefront is the issue of informed consent. Patients undergoing genetic testing should be thoroughly educated about what is being tested, how the results will be used, and the potential implications for their treatment and overall health [148]. Misinterpretation of genetic risk or overemphasis on uncertain data could lead to unnecessary anxiety or overtreatment [149].

Privacy is another major consideration. Genetic information is inherently sensitive and must be protected under stringent data governance frameworks to prevent misuse or unauthorized disclosure [150]. Dental clinics must ensure that patient data is securely stored and that genetic results are used solely for the purpose for which consent was obtained.

Equitable access also poses a challenge. Advanced genetic screening and molecular diagnostics may not be uniformly available across geographic or socioeconomic boundaries [151]. Without careful policy planning, the incorporation of precision dentistry could inadvertently widen healthcare disparities. Universal access to these tools, supported by insurance frameworks or public health programs, will be essential to ensure equitable sharing of benefits [152].

Finally, the successful implementation of genetically informed dentistry hinges on clinician education [153]. Dental professionals must be trained to interpret genetic data, understand its limitations, and communicate findings to patients in a meaningful and ethical way [154]. Continuing education programs and clinical decision support systems will play a vital role in bridging the knowledge gap and fostering responsible integration of these technologies into everyday dental care [155].

## 9. Limitations and Future Directions

Expanding on previously identified methodological and biological limitations, future research in PRR should address critical gaps that impede clinical translation. Much of the currently available evidence is derived from in vitro studies, animal models, or small clinical cohorts, which restricts the generalizability and direct applicability of findings to clinical practice. Additionally, genetic association studies investigating cytokine polymorphisms frequently produce heterogeneous results due to population diversity, environmental influences, and methodological inconsistencies.

A further significant limitation is the absence of standardized protocols for detecting and quantifying cytokine-based biomarkers. While gingival crevicular fluid and saliva are promising diagnostic media, inconsistencies in sampling techniques and analytical methods currently hinder their widespread clinical adoption. Moreover, the predominant focus on individual biomarkers, rather than integrated multi-marker panels, diminishes diagnostic accuracy and predictive utility.

Advancements in PRR management are expected to result from the integration of genomics, bioinformatics, and artificial intelligence. Predictive models that combine genetic, molecular, and clinical data have the potential to transform risk assessment and treatment planning. The creation of multi-marker panels and point-of-care diagnostic tools will further improve early detection and preventive strategies.

Future investigations should emphasize large-scale, longitudinal clinical studies and integrative multi-omics methodologies to validate candidate biomarkers and establish robust predictive signatures. Additionally, examining parallels between PRR and other cytokine-mediated diseases, including osteoporosis, rheumatoid arthritis, cancer-associated bone resorption, and neuroinflammatory disorders, may yield important translational insights. Collectively, these advancements will facilitate the shift toward precision dentistry and align oral health research with broader trends in personalized and translational medicine.

The principal limitations identified in the current literature, together with their potential clinical implications and future research priorities, are summarized in Table 3.

Collectively, the evidence gaps and research priorities outlined in Table 3 emphasize the need for multidisciplinary and translational approaches capable of bridging experimental findings with clinically applicable strategies. These developments may ultimately facilitate the transition toward precision dentistry and personalized patient management.

## 10. Conclusions

Pathological root resorption is primarily a cytokine-mediated condition, wherein inflammatory mediators integrate genetic predisposition, environmental factors, and molecular signaling pathways. Cytokines, including IL-1β, IL-6, and TNF-α, are central to the regulation of osteoclast activity via the RANK/RANKL/OPG axis, while chemokines contribute to sustained cellular recruitment and inflammation.

The dual role of cytokines as mediators and biomarkers places them at the center of emerging diagnostic and therapeutic strategies. Integrating cytokine profiling with genetic and molecular data facilitates the advancement of precision dentistry, enabling early diagnosis, individualized risk assessment, and targeted interventions.

Future research should prioritize the development of cytokine-based diagnostic panels and the therapeutic modulation of inflammatory pathways, aligning dental medicine with broader advances in personalized and translational medicine. Clinically, cytokine profiling is likely to become an essential tool for early detection and personalized treatment planning in patients at risk for PRR.

A comprehensive understanding of cytokine-driven mechanisms in PRR may improve dental outcomes and enhance knowledge of systemic inflammatory diseases, such as osteoporosis, rheumatoid arthritis, and cancer-related bone resorption. In this context, cytokine-focused research could refine current concepts of root resorption pathogenesis and support the broader development of precision medicine for inflammatory diseases.

## Figures and Tables

**Table 1:** Genetic determinants of cytokine-mediated pathways in pathological root resorption (PRR), including evidence strength and methodological considerations.

Gene	Biological Role	Key Polymorphisms	Functional Impact in PRR	Clinical Relevance	Study Type	Strength of Evidence	Key Limitations
IL1B	Pro-inflammatory cytokine signaling	−511C>T	Increased IL-1β expression and osteoclast activation	Early inflammatory response; risk marker for enhanced resorption	Clinical genetic association studies	Moderate	Small cohorts; population variability
IL6	Regulation of inflammatory cascade	−174G>C	Sustained inflammation and osteoclast precursor differentiation	Associated with disease progression and severity	Clinical observational/genetic studies	Moderate	Ethnic variability
TNFA	Amplifies inflammatory signaling	−308G>A	Enhances inflammatory signaling and promotes a pro-resorptive cellular environment	Indicator of increased resorptive activity	Clinical genetic studies	Moderate	Inconsistent replication
TNFSF11 (RANKL)	Osteoclast differentiation	Multiple SNPs	Enhanced RANKL expression and clastic activation	Potential therapeutic target (e.g., anti-RANKL therapy)	Human and experimental studies	High	Limited longitudinal data
TNFRSF11B (OPG)	Inhibits RANKL signaling	Various SNPs	Reduced OPG levels and loss of inhibitory control	Protective factor; imbalance increases PRR susceptibility	Human and experimental studies	High	Small sample sizes
VDR	Immune and bone regulation	FokI, TaqI, BsmI	Modulates inflammatory and bone-remodeling pathways	Gene–environment interaction (vitamin D status); risk stratification	Human observational studies	Moderate	Vitamin D confounding
P2RX7	Osteoclast apoptosis	Loss-of-function variants	Prolonged osteoclast survival and sustained resorption	Potential target for modulating inflammatory responses	Experimental studies	Low–moderate	Limited clinical validation
DSPP	Dentin matrix formation	Various mutations	Structural dentin defects and increased clastic adhesion	Associated with susceptibility to internal resorption	Experimental studies	Low	Mostly mechanistic evidence

Legend: PRR—Pathological Root Resorption; IL1B—Interleukin 1 Beta gene; IL6—Interleukin 6 gene; TNFA—Tumor Necrosis Factor Alpha gene; TNFSF11—Tumor Necrosis Factor Superfamily Member 11 (Receptor Activator of Nuclear Factor Kappa-B Ligand, RANKL); TNFRSF11B—Tumor Necrosis Factor Receptor Superfamily Member 11B (Osteoprotegerin, OPG); VDR—Vitamin D Receptor; P2RX7—Purinergic Receptor P2X 7; DSPP—Dentin Sialophosphoprotein; SNP—Single Nucleotide Polymorphism. Strength of evidence was categorized according to consistency, reproducibility, and translational applicability of the available literature.

**Table 2:** Biomarkers associated with pathological root resorption (PRR), including evidence strength and methodological considerations.

Biomarker	Biological Source	Role in PRR	Diagnostic/ Clinical Utility	Study Type	Strength of Evidence	Key Limitations
IL-1β	GCF, saliva	Promotes osteoclast activation and inflammation	Early marker of inflammatory activity	Clinical studies	High	Lack of standardized thresholds
IL-6	GCF, saliva	Sustains chronic inflammation	Indicator of disease progression	Clinical studies	High	Inter-individual variability
TNF-α	GCF, saliva	Amplifies inflammatory activity and tissue resorption	Marker of resorption severity	Clinical studies	High	Variable analytical methods
MMP-9	GCF, saliva	Degrades extracellular matrix and dentin	Marker of active tissue breakdown	Clinical studies	Moderate–high	Limited specificity
sRANKL	GCF	Reflects osteoclast activation	Correlates with resorptive activity	Human studies	Moderate	Variable collection methods
OPG	GCF	Inhibits osteoclastogenesis	Protective biomarker; low levels indicate risk	Human studies	Moderate	Limited longitudinal evidence
RANKL/OPG ratio	GCF	Reflects balance of bone resorption	Predictive indicator of disease progression	Clinical studies	High	Lack of standard cutoff values
miR-21	Saliva, GCF	Promotes osteoclast differentiation	Potential early predictive biomarker	Experimental + clinical studies	Low–moderate	Limited validation
miR-29b	Saliva, GCF	Regulates bone remodeling pathways	Monitoring tool for resorptive activity	Experimental studies	Low	Mostly experimental data

Legend: PRR—Pathological Root Resorption; GCF—Gingival Crevicular Fluid; IL-1β—Interleukin 1 beta; IL-6—Interleukin 6; TNF-α—Tumor Necrosis Factor alpha; MMP-9—Matrix Metalloproteinase 9; sRANKL—Soluble Receptor Activator of Nuclear Factor Kappa-B Ligand; OPG—Osteoprotegerin; miRNA—MicroRNA. Strength of evidence was categorized according to consistency, reproducibility, and translational applicability of the available literature.

**Table 3:** Current evidence gaps and future research priorities in pathological root resorption (PRR).

Research Area	Current Evidence Gap	Clinical/Scientific Impact	Future Research Priority
Cytokine biomarkers	Small and heterogeneous patient cohorts	Reduced reproducibility and limited clinical implementation	Large-scale longitudinal studies
Genetic susceptibility markers	Population-specific variability in reported associations	Difficult generalization across populations	Multi-center genomic studies involving diverse populations
MicroRNA regulation	Predominantly experimental evidence	Limited translational applicability	Clinical validation and functional studies
Epigenetic mechanisms	Limited mechanistic and longitudinal data	Incomplete understanding of regulatory pathways	Multi-omics integration studies
Biomarker standardization	Variability in sample collection and analytical protocols	Reduced diagnostic reliability	Standardized sampling and analytical methodologies
Diagnostic performance	Limited sensitivity and specificity data	Restricted clinical utility	Development of validated predictive models
Personalized therapeutics	Lack of targeted interventional studies	Insufficient evidence for routine application	Evaluation of biomarker-guided therapeutic strategies
Precision dentistry integration	Limited clinical implementation studies	Delayed translation into routine practice	Integration of genetic, epigenetic, and biomarker-based approaches

Legend: PRR—Pathological Root Resorption.

## Data Availability

No new data were created or analyzed in this study. Data sharing is not applicable to this article.

## Supplementary Materials

The following supporting information can be downloaded at https://www.mdpi.com/article/10.3390/biomedicines14061256/s1. Figure S1: Literature identification and evidence selection process.

## Abbreviations

The following abbreviations are used in this manuscript:

PRR	Pathological Root Resorption
IL-1β	Interleukin-1 beta
IL-6	Interleukin-6
TNF-α	Tumor Necrosis Factor-alpha
RANK	Receptor Activator of Nuclear Factor Kappa-B
RANKL	Receptor Activator of Nuclear Factor Kappa-B Ligand
OPG	Osteoprotegerin
CXCL8	C-X-C Motif Chemokine Ligand 8
IL-8	Interleukin-8
CCL2	C-C Motif Chemokine Ligand 2
MMP-9	Matrix Metalloproteinase-9
GCF	Gingival Crevicular Fluid
sRANKL	Soluble Receptor Activator of Nuclear Factor Kappa-B Ligand
miRNA	MicroRNA
IL1B	Interleukin 1 Beta Gene
IL6	Interleukin 6 Gene
TNFA	Tumor Necrosis Factor Alpha Gene
TNFSF11	TNF Superfamily Member 11
TNFRSF11B	TNF Receptor Superfamily Member 11B
VDR	Vitamin D Receptor
SNP	Single Nucleotide Polymorphism
P2RX7	Purinergic Receptor P2X 7
DSPP	Dentin Sialophosphoprotein
CBCT	Cone-Beam Computed Tomography
ATR-FTIR	Attenuated Total Reflectance Fourier-Transform Infrared Spectroscopy
