# Supplementary material for: Cytokines as Key Drivers of Pathological Root Resorption: Integrating Molecular Mechanisms, Genetic Determinants, and Biomarker-Based Insights
Source: Biomedicines. 2026 May 30;14(6):1256. doi: 10.3390/biomedicines14061256 (PMC13296774; doi:10.3390/biomedicines14061256)
Supplement: Supplementary file 1 [file biomedicines-14-01256-s001.zip › biomedicines-4320283-supplementary.pdf]

Supplementary Figure S1. Literature identification and evidence selection process.

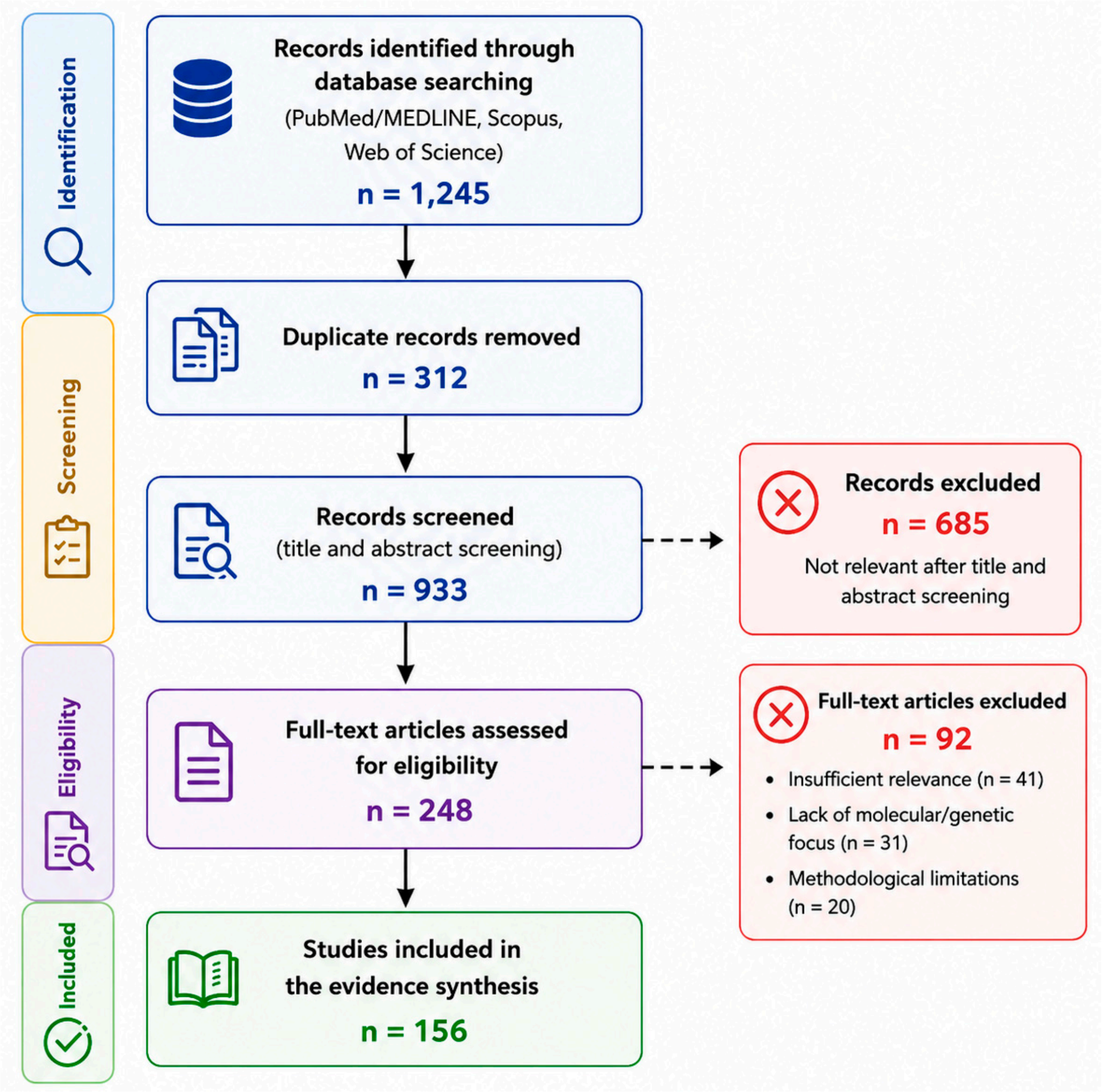

Legend: Flowchart summarizing the literature identification, screening, and evidence selection process used in this integrative review. A total of 1,245 records were identified through database searching. Following duplicate removal, studies underwent title and abstract screening and full-text eligibility assessment before inclusion in the final evidence synthesis.
